# Transcriptome Analysis of Resistance to Fusarium Wilt in Mung Bean (*Vigna radiata* L.)

**DOI:** 10.3389/fpls.2021.679629

**Published:** 2021-06-17

**Authors:** Yujie Chang, Feifei Sun, Suli Sun, Lanfen Wang, Jing Wu, Zhendong Zhu

**Affiliations:** Institute of Crop Sciences, Chinese Academy of Agricultural Sciences, Beijing, China

**Keywords:** mung bean, Fusarium wilt, plant resistance, RNA-seq, WGCNA

## Abstract

Fusarium wilt is a destructive soil-borne disease that threatens the production of mung bean. Mung bean lines Zheng8-4 and Zheng8-20 show high resistance and high susceptibility to Fusarium wilt, respectively. Transcriptome analysis was carried out to identify candidate genes involved in Fusarium wilt resistance using Zheng8-4 and Zheng8-20 at 0, 0.5, 1, 2, and 4 days post inoculation (dpi). Differential expression analysis showed that 3,254 genes responded to pathogen infection and were differentially expressed in the resistant and susceptible lines. Weighted gene co-expression network analysis (WGCNA) was also performed to identify five modules highly correlated with Fusarium wilt resistance, in which 453 differentially expressed genes (DEGs) were considered likely to be involved in Fusarium wilt resistance. Among these DEGs, we found 24 genes encoding resistance (R) proteins, 22 encoding protein kinases, 20 belonging to transcription factor families, 34 encoding proteins with oxidoreductase activity, 17 involved in stimulation/stress responses, and 54 annotated to pathogen resistance-related pathways. Finally, 27 annotated genes were further selected as candidate genes of Fusarium wilt resistance in mung bean. This study identifies novel potential resistance-related genes against Fusarium wilt and provides a theoretical basis for further investigation of Fusarium wilt resistance in mung bean breeding.

## Introduction

Mung bean (*Vigna radiata* L.) is an economically important grain legume crop used for human consumption. It is cultivated mostly in South, East and Southeast Asia for its edible seeds and sprouts (Schafleitner et al., 2015). Mung bean seeds are a good source of dietary protein and contain high levels of folate and iron (Keatinge et al., 2011). Moreover, intercropping mung bean with various crops increases the yield of subsequent cereal crops and reduces pest incidence because, as a legume, mung bean can improve soil fertility by fixing atmospheric nitrogen (Graham and Vance, 2003).

The productivity and quality of mung bean are largely limited by various biotic stresses, and disease is among the main factors. Among these diseases, Fusarium wilt has become increasingly severe in most mung bean cultivation areas (Sun et al., 2019). Fusarium wilt is a destructive soil-borne disease caused by *Fusarium oxysporum. F. oxysporum* strains have a broad host range and have been divided into various *formae speciales* according to their host specificity (Sun et al., 2019). This pathogen causes root rot, vascular wilt, and leaf wilt, with stunted growth and eventual death (Choudhary et al., 2017). It is difficult to manage the disease either through crop rotation or application of fungicides because of its soil-borne nature (Edel-Hermann and Lecomte, 2019). Therefore, developing resistant cultivars is considered to be the most effective measure to control Fusarium wilt (Manzo et al., 2016; Upasani et al., 2017).

A variety of defence mechanisms in plant-*F. oxysporum* interactions have been studied in several species, such as tomato (Manzo et al., 2016), sesame (Wei et al., 2016), cotton (Dowd et al., 2004), soybean (Lanubile et al., 2015), and common bean (Xue et al., 2015). After Fusarium wilt infection, plants usually undergo a series of changes in physiology, biochemistry and molecular biology, including wound responses, hypersensitive reactions, and gene expression and metabolic changes (Chen et al., 2019). It has been reported that Fusarium wilt resistance is related to the resistance (R) gene, protein kinases and some oxidoreductases. The leucine-rich repeat (LRR) is the most commonly conserved domain of R proteins. Nucleotide-binding domain and leucine-rich repeat (NLR) containing protein I-2, leucine-rich repeat receptor-like proteins (LRR-RLP) I-7 and Ve1 were identified in tomato and confer resistance to Fusarium wilt resistance (Fradin et al., 2009; Giannakopoulou et al., 2015; Gonzalez-Cendales et al., 2016). The tomato gene *I-3* encodes an S-receptor-like kinase that was also identified to be involved in resistance to Fusarium wilt disease (Catanzariti et al., 2015). A number of protein kinases were also predicted to be related to Fusarium wilt resistance in melon, chickpea, and common bean (Sebastiani et al., 2017; Upasani et al., 2017; Chen et al., 2019). Moreover, a peroxidase gene (*PvPOX1*) of common bean was proven to be upregulated by *F. oxysporum* infection, and overexpression of *PvPOX1* in hairy roots could increase resistance to Fusarium wilt (Xue et al., 2014, 2017). In addition, transcription factors also play important roles in the responses to pathogen infections by regulating the expression of pathogen-responsive genes (Amorim et al., 2017). Some transcription factors were found to respond to Fusarium wilt infections (Manzo et al., 2016; Sebastiani et al., 2017; Zhang et al., 2019). Furthermore, plant defence can be induced by salicylic acid (SA), jasmonic acid (JA), defence metabolites (phenolic acids, polyphenols, and flavonoids), etc (Sebastiani et al., 2017; Zhou and Zhang, 2020). Secondary metabolites were also reported to play roles in the response to *F. oxysporum* infection in common bean (Chen et al., 2019, 2020).

As most plant defence responses can be observed at the transcriptional level, transcriptome analyses can provide insights into the type of defence mechanism involved in the Fusarium wilt disease reaction. The plant-*F. oxysporum* interaction has been studied by transcriptome analysis in many important horticultural and agricultural crops. Transcriptional changes were investigated in resistant (*Momor*) and susceptible (*Monalbo*) isogenic tomato lines after infection by *F. oxysporum*, and a key gene (*CYP83B1*) was found to be related to tomato-pathogen interactions (Manzo et al., 2016). RNA-Seq analysis was also used to identify candidate resistance genes during the melon-*F. oxysporum* race 1.2 interaction at 24 and 48 h post inoculation (hpi) in resistant and susceptible genotypes (Sebastiani et al., 2017). In banana, transcriptomic analysis was performed using highly resistant and susceptible cultivars, showing that some DEGs were thought to play key roles in the response to *F. oxysporum* at the early infection stage (Zhang et al., 2019). Furthermore, plant-*F. oxysporum* interactions, as revealed by transcriptome analyses, have also been reported for some legumes. Using RNA-seq, the interactions of a partially resistant soybean genotype and the non-pathogenic/pathogenic isolates of *F. oxysporum* were observed and found to include many defence-related genes, transcription factors and several genes involved in ethylene biosynthesis and secondary and sugar metabolism (Lanubile et al., 2015). In chickpea, several genes involved in the interactions between chickpea and *F. oxysporum* were identified via transcriptome profiling and were related to lignification, plant defence signalling, hormonal homeostasis, R gene-mediated defence, etc (Upasani et al., 2017). In view of these findings, transcriptome analysis can be considered an effective method for studying plant-*F. oxysporum* interactions and selecting resistance-related genes for Fusarium wilt.

In mung bean, few studies have been conducted to understand the molecular mechanism involved in the response to Fusarium wilt infection. The defence mechanisms underlying resistance to Fusarium wilt remain unclear. In the present study, we selected highly resistant (Zheng8-4) and highly susceptible (Zheng8-20) lines to investigate transcriptome alterations after Fusarium wilt infection. Differential expression analysis and weighted gene co-expression network analysis (WGCNA) were carried out to identify putative resistance-related genes that responded to Fusarium wilt infection. This study provides important insights for breeding resistant cultivars and investigating the resistance mechanisms against Fusarium wilt in mung bean.

## Materials and Methods

### Plant Materials and Fungal Strain

Zhenglv8 is an excellent mung bean cultivar planted in Henan Province, China. Mung bean lines Zheng8-4 and Zheng8-20, segregated from Zhenglv8, were obtained from resistance screening and continued to be screened for several years, showing resistance and susceptibility to Fusarium wilt, respectively. *F. oxysporum* strain F08 was isolated from a naturally infected mung bean plant. Strain F08 was routinely cultivated in Petri dishes containing potato dextrose agar (PDA) at 25°C.

### Plant Infection and *F. oxysporum* Resistance Evaluation

The inoculation was performed as described previously (Sun et al., 2019). The seeds that were planted in the paper cups were grown in the greenhouse for 2 weeks at 22 ∼ 25°C until the primary leaves of the seedlings had fully expanded. The seedlings were inoculated with *F. oxysporum* though the root cutting technique. Fifteen inoculated plants were then transplanted into a new cup, and three replicate cups were performed for each line. The symptoms were investigated 2 weeks after inoculation. Disease severity was scored based on the average severity of the 15 plants. All pathogenicity tests were repeated three times.

### RNA Sequencing

The roots of 15 plants per time point were pooled and used for transcriptome analysis. Three replicates of five time points (0, 0.5, 1, 2, and 4 dpi) from Zheng8-4 and Zheng8-20 were collected. A total of 30 RNA samples were extracted by TRIzol reagent (Invitrogen, Carlsbad, United States) according to the manufacturer’s instructions. Illumina sequencing was performed at Novogene, China. Sequencing libraries were generated using the NEBNext Ultra^TM^ RNA Library Prep Kit for Illumina (NEB, Ipswich, MA, United States). The library preparations were sequenced on an Illumina HiSeq 2500 platform, and 150 bp paired-end reads were generated. Clean reads were obtained by removing reads containing adapters, reads containing poly-N and low-quality reads from raw data. The Q20, Q30, and GC contents of the clean data were calculated. All raw-sequence reads data were stored on the NCBI SRA platform with accession number PRJNA715596.

Reference genome and gene model annotation files were downloaded from http://plants.ensembl.org/Vigna_radiata/Info/Index. The paired-end clean reads were aligned to the reference genome using Hisat2 v2.0.5. FeatureCounts v1.5.0-p3 was used to count the read numbers mapped to each gene. All unigenes were annotated by alignment to seven public databases, including the Nr, Nt, Pfam, Swiss-Prot protein, Clusters of orthologous groups for eukaryotic complete genomes/Clusters of Orthologous Groups (KOG/COG), Gene Ontology (GO), and Kyoto Encyclopedia of Genes and Genomes (KEGG) databases. Novel transcript prediction was performed using StringTie (v1.3.3b). The expected number of fragments per kilobase of transcript sequence per million base pairs sequenced (FPKM) of each gene was calculated based on the length of the gene and read count mapped to the gene.

### Differential Expression Analysis

Differential expression analysis was performed using the DESeq2 R package 1.16.1. The resulting *p*-values were adjusted using the Benjamini and Hochberg method. Genes with an adjusted *p*-value < 0.05 and fold change > 2 were assigned as differentially expressed genes/transcripts. Bar and Venn diagrams were created with the R package.

GO enrichment analysis of differentially expressed genes was performed by the clusterProfiler R package. GO terms with corrected *p* < 0.05 were assigned as significantly enriched. KEGG pathway enrichment analysis of the DEGs was implemented by KOBAS 3.0^1^.

### Gene Co-expression Network Analysis

Gene co-expression networks were constructed using the WGCNA package with R software (version 3.6.1). The unigenes with very low expression (FPKM < 1) in all samples were removed from this analysis to avoid the inclusion of spurious edges in the networks. Gene modules were identified using the parameter β value 7 (Supplementary Figure 1), minimum cluster size 30, and merging threshold function 0.25. Gene significance (GS) was used to correlate physiological data with the gene expression data.

### Quantitative Real-Time PCR Analysis

A total of 27 DEGs were selected to validate the results of the RNA-seq by quantitative real-time PCR (qRT-PCR). The UEIrisII RT-PCR System (BioDee, Beijing, China) was used to synthesize first-strand cDNA. The housekeeping gene *CYP2* was used as the internal reference gene (Jian et al., 2008; Li et al., 2015). The gene-specific primers were designed using Primer 3 (v. 0.4.0) (Supplementary Table 1). qRT-PCR was conducted using an ABI 7500 Real-time System (Applied Biosystems, United States). qRT-PCR was performed using 150 ng of cDNA, 10 μL of Top Green qPCR SuperMix (TransGen, Beijing, China) and 0.4 μM of each primer in a 20 μL volume under the following conditions: enzyme activation for 30 s at 94°C, followed by 40 cycles of 94°C for 5 s, 58°C for 15 s, and 72°C for 34 s. The qRT-PCR experiment was repeated with three biological replicates for each sample. The relative expression of target genes was calculated with the 2^ΔΔ*CT*^ method (Schmittgen and Livak, 2008).

## Results

### Phenotypic Differences Between the Resistant and Susceptible Lines

**FIGURE 1:**
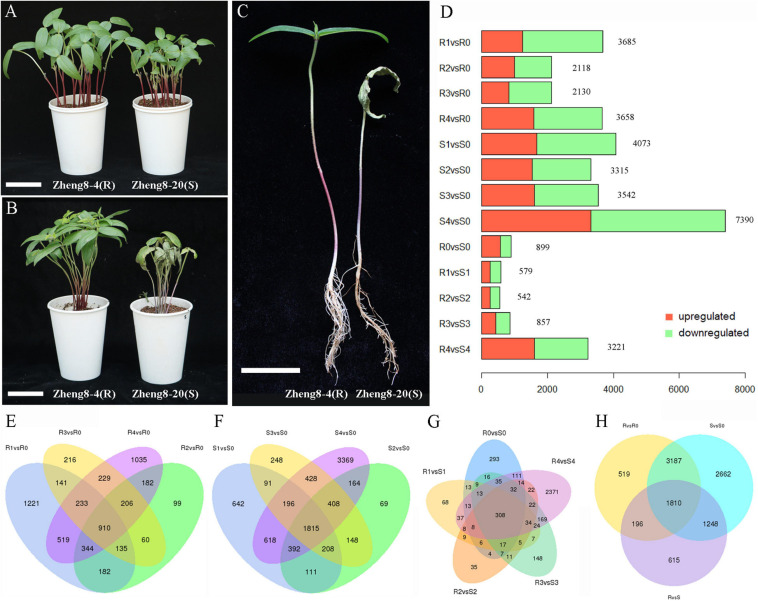
Symptoms of Fusarium wilt and differential expression analysis in the resistant line (Zheng8-4) and the susceptible line (Zheng8-20). **(A)** The phenotype of two lines at 0 dpi. **(B,C)** The symptoms of Fusarium wilt at 14 dpi. Bars = 5 cm. **(D)** Number of differentially expressed genes (DEGs) in each pair. Red, upregulated DEGs; Green, downregulated DEGs. **(E,F)** Venn diagram of the DEGs before and after inoculation in the resistant **(E)** and susceptible lines **(F)**. **(G)** Venn diagram of the DEGs between the resistant and susceptible lines. **(H)** Venn diagram of the DEGs in the two DEG analyses. R, resistant line Zheng8-4; S, susceptible line Zheng8-20; 0–4, 0, 0.5, 1, 2, and 4 dpi, respectively.

Mung bean lines Zheng8-4 and Zheng8-20 were separated from the Zhenglv8 cultivar, which is widely bred in China. Two lines showed significantly different resistance to Fusarium wilt: Zheng8-4 was resistant to Fusarium wilt, and Zheng8-20 was susceptible. The Fusarium wilt resistance test was performed using these two lines infected with *F. oxysporum* F08. Zheng8-20 plants started to show Fusarium wilt symptoms of leaf yellowing and plant wilt at 7 dpi. After 14 dpi, all Zheng8-4 plants remained healthy and free of wilt symptoms, while all inoculated Zheng8-20 plants showed severe wilt symptoms, including leaf and stem wilt, root rot, and plant death (Figures 1A–C). Plant growth of susceptible line also hindered by Fusarium wilt, such as plant height reduction. These results confirmed that the Zheng8-4 line was highly resistant to Fusarium wilt and that the Zheng8-20 line was highly susceptible to Fusarium wilt.

### Transcriptome Sequencing and Assembly

To investigate genes responding to Fusarium wilt resistance in mung bean, transcriptome sequencing was carried out using Zheng8-4 (resistant line) and Zheng8-20 (susceptible line) at five time points (0, 0.5, 1, 2, and 4 dpi). A total of 1,265,802,600 clean reads were generated, and each sample yielded approximately 6 Gb clean reads (Supplementary Table 2). The Q20 and Q30 percentages were more than 98.14% and 94.39%, respectively. The average GC content of the transcriptome was 44.05% (Supplementary Table 2). Mapping the clean reads to the reference genome (Kang et al., 2014) showed that an average of 95.34% of reads were mapped in total, and 93.46% were uniquely mapped (Supplementary Table 2). A total of 26,716 unique genes were identified.

The expression values of all these genes were determined by the expected FPKM values. To verify the reproducibility of the sequencing data, Pearson’s correlation coefficients for three biological replicates were calculated by log_10_(FPKM + 1). All biological replicates had a strong correlation (*R*^2^ ≥ 0.9) (Supplementary Figure 2 and Supplementary Table 3). Moreover, principal component analysis (PCA) revealed that the samples of the same treatment were more clustered and that differences existed between resistant and susceptible lines (Supplementary Figure 2). A total of 21,964 genes with an FPKM ≥ 1 in at least one of these samples were considered expressed and subjected to further analysis.

### Identification of Differentially Expressed Genes

To identify DEGs that responded to Fusarium wilt infection, transcriptome comparisons were conducted for two lines before and after inoculation (0.5 dpi vs. 0 dpi, 1 dpi vs. 0 dpi, 2 dpi vs. 0 dpi, and 4 dpi vs. 0 dpi). Using a significance level of fold change > 2 and *p*-value ≤ 0.05, a total of 5,712 DEGs were found in Zheng8-4 at different time points after inoculation (0.5, 1, 2, and 4 dpi) compared to those at 0 dpi, and 3,141 DEGs were found in two or more compared pairs (Figures 1D,E and Supplementary Table 4). In Zheng8-20, a total of 8,907 DEGs were identified, including 4,579 DEGs found in more than one compared pair (Figures 1D,F and Supplementary Table 4). Combining the DEGs in Zheng8-4 and Zheng8-20, a total of 9,622 DEGs were identified to respond to pathogen infection. A total of 4,997 DEGs responded to *F. oxysporum* infection in both cultivars (Figure 1H). Among these pathogen-responsive DEGs, differences existed in the timing and expression patterns of pathogen response but also between resistant and susceptible lines.

To further identify genes putatively involved in the resistance response, differential expression analyses were performed between resistant and susceptible lines at five time points. There were 899, 579, 542, 857, and 3,221 DEGs found between Zheng8-4 and Zheng8-20 at 0, 0.5, 1, 2, and 4 dpi, respectively (Supplementary Table 5). A total of 3,869 DEGs involved in the resistance response were identified, 954 of which were repeatedly identified in two or more comparison groups, and most DEGs were found at 4 dpi (Figure 1G).

Considering 3,869 resistance-response DEGs and 9,622 pathogen-responsive DEGs identified above, 3,254 DEGs were selected that were identified in both of the two DEG analyses (Figure 1H). These 3,254 DEGs responded to pathogen infection and were differentially expressed in the resistant and susceptible lines, implying their putative involvement in Fusarium wilt resistance in mung bean.

**FIGURE 2:**
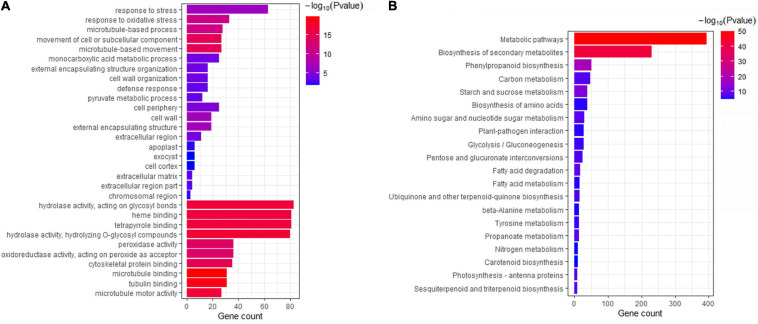
Functional analysis of 3254 DEGs. **(A)** GO enrichment of DEGs. **(B)** KEGG enrichment of DEGs.

To evaluate the possible function of the 3,254 DEGs, GO enrichment was applied to identify the major functional categories. These DEGs were differentially enriched in 729 terms (Supplementary Table 6). The terms were dominant within the biological process, molecular function and cellular component categories, such as “response to stress,” “response to oxidative stress,” “cell periphery,” “cell wall,” “hydrolase activity,” and “tetrapyrrole binding” (Figure 2A and Supplementary Table 6). Additionally, these DEGs were mapped to 125 KEGG pathways (Supplementary Table 7). Most DEGs were significantly enriched in “metabolic pathways” and “biosynthesis of secondary metabolites” (Figure 2B). Some genes were significantly clustered into pathogen response-related pathways, such as “plant-pathogen interaction,” “phenylpropanoid biosynthesis,” and “pentose and glucuronate interconversions” (Figure 2B and Supplementary Table 7). These results provide a functional reference for further screening Fusarium wilt resistance-related genes in mung bean.

### Identification of Co-expression Network Modules

**FIGURE 3:**
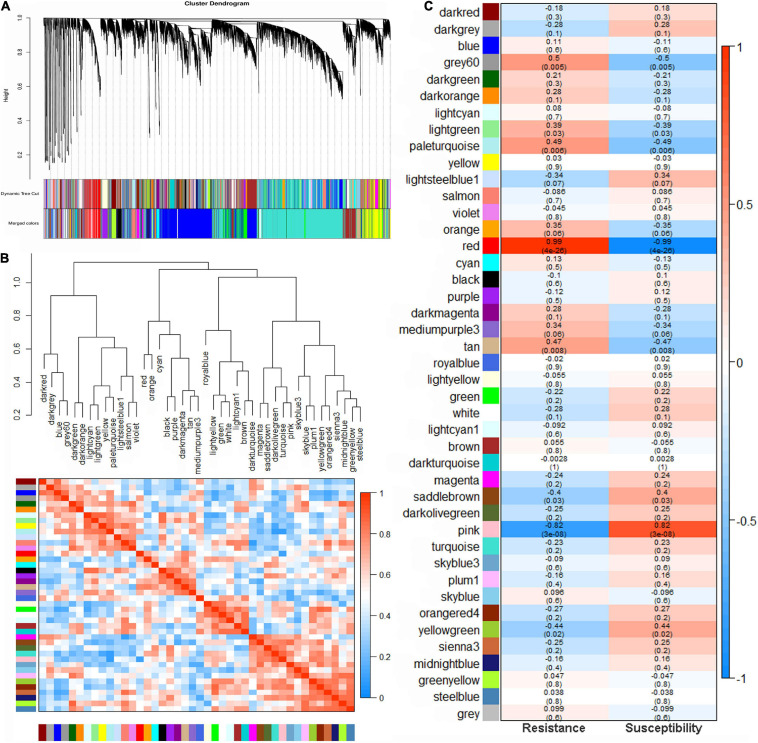
Weighted gene co-expression network analysis (WGCNA) in mung bean infected with Fusarium wilt. **(A)** Hierarchical cluster diagram of co-expression modules according to WGCNA. **(B)** Gene co-expression modules showing the cluster dendrogram (above) and the heatmap for the correlation coefficient between the modules (below). **(C)** Module–trait relationships. Each cell contains the corresponding correlation and *p*-value.

To further identify genes related to pathogen resistance in mung bean, highly co-expressed gene modules were inferred from all genes using WGCNA. A total of 21,091 genes were selected to construct the network after removing genes with very low expression (an average FPKM of <1). When the power value (β value) was 7, 42 modules with module sizes ranging from 35 to 5,510 genes were generated, and the 170 genes outside any module were clustered into a grey module (Figure 3A and Supplementary Table 8). The genes of each module were considered to be co-expressed and functionally related. The heatmap of correlations among these modules indicated that the high correlation modules with red squares may be involved in a similar biological process (Figure 3B).

### Potential Genes Related to Fusarium Wilt Resistance in Mung Bean

**FIGURE 4:**
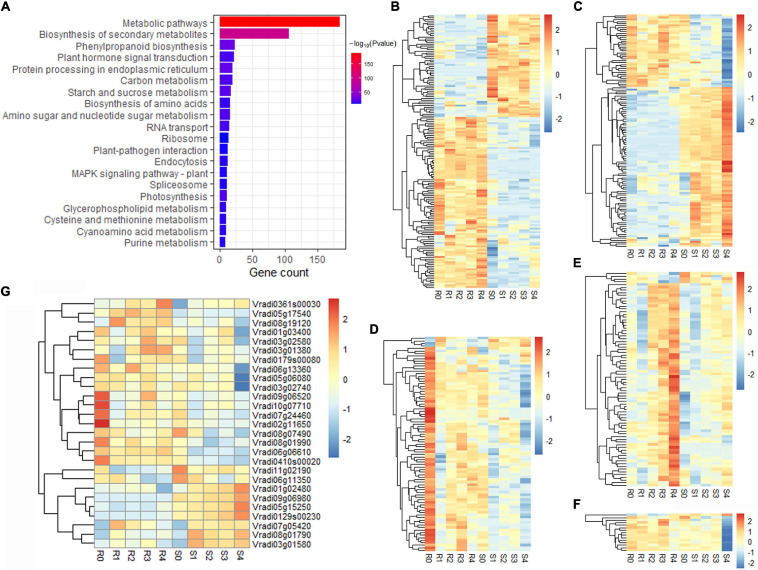
Functional and expression pattern analyses of DEGs in five modules (red, pink, grey60, tan, and paleturquoise) related to Fusarium wilt resistance. **(A)** KEGG enrichment of DEGs in five modules related to Fusarium wilt resistance. **(B-F)** Expression patterns of co-expressed genes in the red **(B)**, pink **(C)**, grey60 **(D)**, tan **(E)**, and paleturquoise **(F)** modules, respectively. **(G)** Expression patterns of 27 candidate genes.

Five modules were significantly (*p* < 0.01) correlated with *F. oxysporum* resistance: the red (*R* = 0.99, *p* = 4e^–26^), grey60 (*R* = 0.50, *p* = 0.005), paleturquoise (*R* = 0.49, *p* = 0.006) and tan (*R* = 0.47, *p* = 0.008) modules showed a positive correlation, indicating that genes in these correlated modules positively regulate pathogenic resistance, while the pink (*R* = −0.82, *p* = 3e^–08^) module had a negative correlation, suggesting that the genes in the module negatively regulate pathogenic resistance (Figure 3C). A total of 886, 466, 245, 297, and 78 genes were found in the red, pink, grey60, tan, and paleturquoise modules, respectively (Supplementary Table 8). KEGG pathway enrichment analysis of the genes in the five correlation modules showed that genes were significantly clustered into pathogen resistance-related pathways, such as “phenylpropanoid biosynthesis,” “plant hormone signal transduction,” “plant-pathogen interaction,” and “MAPK signalling pathway” (Figure 4A and Supplementary Table 9), confirming the high correlation between these five modules and pathogen resistance.

On the basis of the results of differential expression analysis and the co-expression network, 3,254 DEGs were clustered into 36 modules, and 51 DEGs outside any modules were grouped into the grey module. A total of 453 DEGs were identified in the five resistance-correlated modules. There were 155, 119, 85, 79, and 15 DEGs grouped into the red, pink, grey60, tan and paleturquoise modules, respectively (Supplementary Table 8). The majority of DEGs in the red and pink modules had completely opposite expression patterns in resistant and susceptible lines (Figures 4B,C); most DEGs in the grey60 module were dominantly expressed in the resistant line before inoculation, and their expression levels were decreased after inoculation in both lines (Figure 4D); in the tan module, the DEGs were dominantly expressed in the resistant line at 4 dpi (Figure 4E); in the paleturquoise module, the expression of DEGs was significant decreased in the susceptible line at 4 dpi (Figure 4F). These 453 DEGs may be associated with Fusarium wilt resistance and used for further analysis.

Among the 453 DEGs, a total of 24 R genes and 22 DEGs encoding protein kinases were identified (Supplementary Table 10). Twenty transcription factor genes were obtained in the resistance-correlated modules, belonging to the MADS, MYB, WRKY, AP2, NAC, HD-ZIP gene families, etc (Supplementary Table 10). Moreover, we also found 34 DEGs with oxidoreductase activity, 9 of which were annotated as cytochrome P450 genes, 5 as peroxidase genes and 20 as other oxidoreductases (Supplementary Table 10). Additionally, GO analysis showed that 17 DEGs were annotated with the term “response to stimulus” or “response to stress”, indicating that these genes may respond to Fusarium wilt infection (Supplementary Table 10). KEGG analysis also found that 15 DEGs were annotated against pathogen resistance-related pathways, including “plant-pathogen interaction,” “MAPK signalling pathway,” “plant hormone signal transduction,” and “endocytosis” (Supplementary Table 10). We also found 39 DEGs associated with the biosynthesis of secondary metabolites, such as “phenylpropanoid biosynthesis” and “terpenoid biosynthesis” (Supplementary Table 10). The functional annotations suggested that these DEGs may be related to Fusarium wilt resistance in mung bean.

### Identification and Characterisation of Candidate Genes

The candidate resistance-related genes were further screened from 453 DEGs by the following criteria: (1) genes were annotated in the reference genome, and one or more annotations were related to pathogen resistance; and (2) gene expression patterns were significantly different between resistant and susceptible lines. Finally, 27 candidate genes were selected for Fusarium wilt resistance of mung bean, including seven R genes, seven protein kinase genes, six transcription factor genes and seven oxidoreductase genes (Supplementary Table 10). Functional analysis revealed that two R genes (*Vradi09g06980* and *Vradi07g05420*), three peroxidase genes (*Vradi06g06610*, *Vradi11g02190*, and *Vradi07g24460*) and one plant hormone responsive gene (*Vradi05g15250*) responded to stimulation or stress. The R gene *Vradi03g02580*, transcription factor gene *Vradi03g01580* and oxidase gene *Vradi05g06080* were involved in the “MAPK signalling pathway”. *Vradi01g02480* and *Vradi10g07710* were associated with “plant-pathogen interactions.” Among these, 18 genes showed higher transcript accumulation in the resistant line than in the susceptible line, implying their positive roles in Fusarium wilt resistance (Figure 4G). In contrast, nine genes were highly expressed in the susceptible line, showing that these genes may negatively regulate Fusarium wilt resistance (Figure 4G). All of these candidate genes were predicted to be related to Fusarium wilt resistance and should be prioritised in further investigation.

**FIGURE 5:**
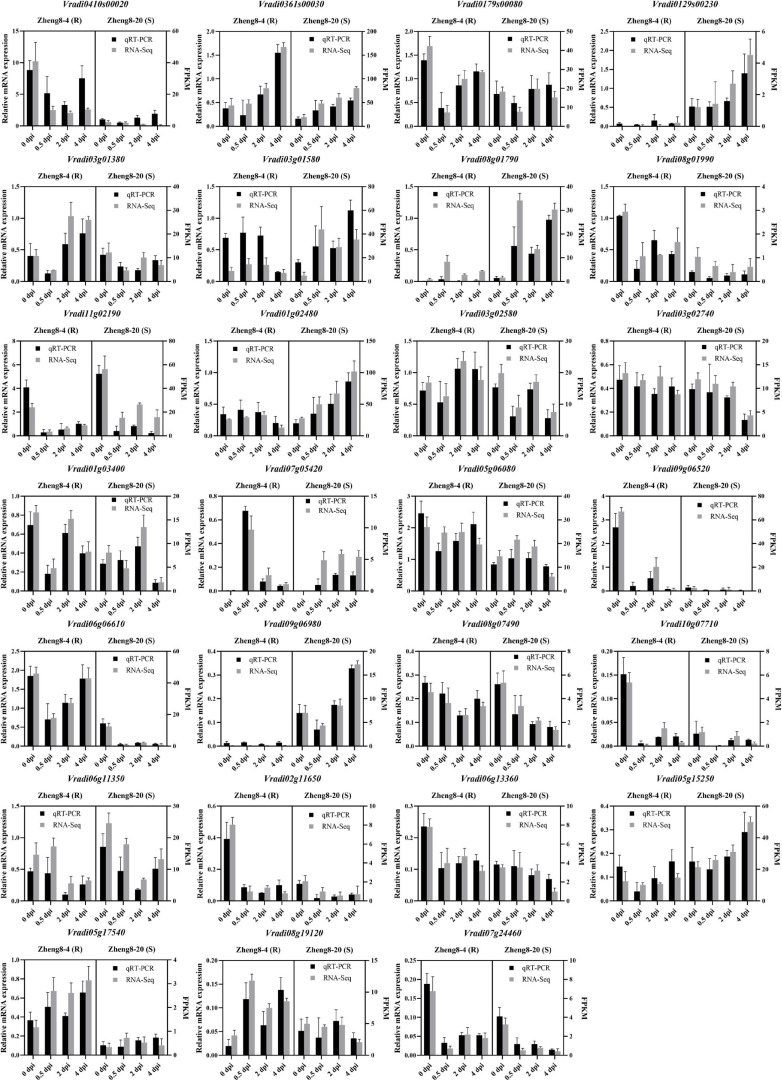
qRT-PCR validation of the selected DEGs in mung bean. R, resistant line; S, susceptible line; dpi, day post inoculation.

To validate the RNA-seq data results, 27 candidate DEGs were selected for validation of the transcriptomic data using qRT-PCR. The expression trend of 26 genes in qRT-PCR analysis was consistent with the RNA-seq data, while only the result of *Vradi0410s00020* slightly differed from the corresponding RNA-seq data (Figure 5 and Supplementary Table 11), which supported the results generated by RNA-seq. This result confirmed the reliability of the RNA-seq data in the present study. Additionally, it is notice that the differences of genes expression between resistant and susceptible lines. The expression of 14 candidate genes (*Vradi0179s00080*, *Vradi01g03400*, *Vradi02g11650*, *Vradi0361s00030*, *Vradi03g01580*, *Vradi0410s00020*, *Vradi05g06080*, *Vradi05g17540*, *Vradi06g06610*, *Vradi06g13360*, *Vradi07g24460*, *Vradi08g01990*, *Vradi09g06520*, and *Vradi10g07710*) showed higher expression in resistant line at 0 dpi. Four candidate genes (*Vradi0129s00230*, *Vradi06g11350*, *Vradi09g06980*, and *Vradi11g02190*) were dominantly expressed in susceptible line at 0 dpi (Figures 4G, 5 and Supplementary Table 11). Especially, *Vradi09g06520* was dominantly expressed in resistance line and extremely low expressed in susceptible line. *Vradi0129s00230* and *Vradi09g06980* almost not expressed in resistance line (Figures 4G, 5 and Supplementary Table 11). The original expression differences of these genes between resistant and susceptible lines may affect resistant response in mung bean.

## Discussion

In the present study, transcriptome analysis of Fusarium wilt resistance was performed using two mung bean lines, Zheng8-4 and Zheng8-20. These two lines showed diametrically opposite phenotypes after Fusarium wilt inoculation: 100% of inoculated Zheng8-4 plants were resistant to Fusarium wilt, and 100% of inoculated Zheng8-20 plants were susceptible and plant growth hindered by Fusarium wilt, such as plant height reduction (Figures 1A–C). This result showed that the resistance of Fusarium wilt in Zheng8-4 should be a qualitative characteristic, which is more suitable for screening resistance-related genes though transcriptome analysis. Transcriptome analysis is an effective way to study plant-pathogen interactions. Plant-*F. oxysporum* interactions have been studied by transcriptome analysis in banana (Zhang et al., 2019), melon (Sebastiani et al., 2017), sesame (Wei et al., 2016), tomato (Manzo et al., 2016), cabbage (Xing et al., 2016) and some legume crops, such as soybean (Lanubile et al., 2015), chickpea (Upasani et al., 2017) and common bean (Chen et al., 2019). Additionally, WGCNA is a reliable and effective method for the analysis of gene functions and has been successfully used in studying plant responses during pathogen infections. For example, the key genes that responded to *Phytophthora infestans*, *Ralstonia solanacearum*, and *potato virus Y* infections in potato were obtained using WGCNA (Cao et al., 2020). Therefore, transcriptome analysis and WGCNA were carried out to identify putative genes related to Fusarium wilt resistance using two mung bean lines in our study. Compared with transcriptomic studies of other legume, our studies focus on transcriptome analysis of *F. oxysporum* response at both early and late stages after pathogenic infection, and WGCNA is first used to screen Fusarium wilt resistance-related genes. The resistance-related genes in mung bean contribute to R-gene mediated defence, signal transduction, structural defence, secondary metabolites, stress response, etc., which is consistent with previous studies in legume (Lanubile et al., 2015; Upasani et al., 2017; Chen et al., 2019).

Pathogen attack usually affects the structure and integrity of the plant cell wall (Zhang et al., 2020). In the present study, some resistance-related DEGs were significantly associated with cell wall organisation and modification pathways, such as “cell wall organisation,” “cell wall,” and “cell wall modification,” confirming that the genes related to structural defences responded to Fusarium wilt infection in mung bean (Figure 2 and Supplementary Table 6). Cytoskeletal remodelling is considered to play a key role in signal transduction of the plant defence response (Lian et al., 2021). We also found that some DEGs were related to cytoskeletal remodelling and clustered into “microtubule-based movement,” “microtubule-based process,” and “cytoskeletal protein binding” (Figure 2 and Supplementary Table 6), implying that Fusarium wilt affected defence signal transduction in mung bean. Additionally, *F. oxysporum* resistance is related to some metabolites (Lanubile et al., 2015; Chen et al., 2019). A number of resistance-related DEGs were also annotated to the biosynthesis of secondary metabolites (Figure 2 and Supplementary Table 7). These results revealed the function of Fusarium wilt resistance-related genes in mung bean, which is consistent with previous studies and confirmed the reliability of our results.

The 27 candidate genes identified from 453 DEGs may be related to Fusarium wilt resistance. Among them, five R genes (*Vradi03g02580*, *Vradi03g02740*, *Vradi06g11350*, *Vradi07g05420*, and *Vradi08g07490*) were homologous to the Fusarium wilt R genes of tomato and *Brassica oleracea* (Catanzariti et al., 2015; Giannakopoulou et al., 2015; Shimizu et al., 2015; Gonzalez-Cendales et al., 2016), implying their potential roles in Fusarium wilt resistance in mung bean. *Vradi03g02580* and *Vradi08g07490* were homologous to the Fusarium wilt R gene *I-7* in tomato. *I-7* was identified as a gene encoding a leucine-rich repeat receptor-like protein that conferred Fusarium wilt resistance in tomato (Gonzalez-Cendales et al., 2016). *Vradi03g02580* was also associated with the “MAPK signalling pathway”, a major early signalling pathway required for disease defences (Supplementary Table 10). *Vradi06g11350* and *Vradi03g02740* encode leucine-rich repeat receptor kinases (LRR-RKs) and homologous to the R gene *I-3* of tomato. *I-3* is an S-receptor-like kinase gene that confers *Avr3*-dependent resistance to *F. oxysporum* (Catanzariti et al., 2015). *Vradi07g05420* was identified as a NLR gene involved in response to stimuli (Supplementary Table 10), and it is homologous to the R gene *FocBo1*, which is involved in resistance to *F. oxysporum* infection in *Brassica oleracea* (Shimizu et al., 2015). These results implied that these genes may have a similar function to their homologous genes conferring Fusarium wilt resistance. Three putative R proteins (Vradi01g03400, Vradi02g11650, and Vradi09g06980) and three protein kinases (Vradi05g17540, Vradi08g01790, and Vradi0410s00020) were also selected because of their significant differences between the resistant and susceptible lines (Figure 4G). Additionally, several R genes were differently expressed between resistant and susceptible lines before pathogen infection, which may play a role in Fusarium wilt resistance. For example, NLR gene *Vradi02g11650* showed higher expression in resistant line than susceptible line at 0 dpi; another NLR gene *Vradi09g06980* almost not expressed in resistance line and dominantly expressed in susceptible line (Figures 4G, 5 and Supplementary Table 11). The expressed difference of NLRs may affect pathogen identification and immune activation in mung bean.

Peroxidase is an important oxidoreductase in plant defence responses involved in reactive oxygen species (ROS) generation and cell wall modifications (Mittler et al., 2004; Passardi et al., 2004). The peroxidase gene *PvPOX1* has been reported to enhance Fusarium wilt resistance in common bean (Xue et al., 2017). Three peroxidase genes (*Vradi06g06610*, *Vradi11g02190*, and *Vradi07g24460*) that responded to stimulus/stress were selected for further investigation (Supplementary Table 10). Vradi06g06610 and Vradi07g24460 were also annotated to the resistance-related pathway “phenylpropanoid biosynthesis” (Supplementary Table 10). Cytochrome P450 was reported to play roles in the response to *F. oxysporum* infection in tomato (Manzo et al., 2016). In this study, Vradi03g01380 and Vradi0179s00080 were predicted to be cytochrome P450s and dominantly expressed in the resistant line (Figure 4G), implying their putative positive roles in the biosynthesis of metabolites related to disease defence. An oxidase (Vradi05g06080) was clustered into the “plant-pathogen interaction” and “MAPK signalling pathway” terms (Supplementary Table 10), and it may also be associated with Fusarium wilt resistance.

Transcription factors are considered to play key roles in pathogen infections by regulating defence-related signalling pathways (Amorim et al., 2017; Zhou and Zhang, 2020). For example, *Arabidopsis* MYB30 responds to ROS signalling during defence responses (Mabuchi et al., 2018). Vradi0129s00230 is an MYB transcription factor and was expressed only in the susceptible line (Figure 4G), implying that it may negatively regulate Fusarium wilt resistance. We also found three ethylene-responsive transcription factors, Vradi03g01580, Vradi08g19120, and Vradi06g13360, belonging to the AP2 family. Vradi03g01580 was also related to the “MAPK signalling pathway” (Supplementary Table 10).

Moreover, auxin also influences pathogen resistance together with salicylic acid, jasmonic acid, and ethylene (Lee et al., 2009; Deng et al., 2020). Vradi05g15250 is an auxin-responsive protein that responds to stimuli/hormones and participates in “plant hormone signal transduction” (Supplementary Table 10). Its expression increased after *F. oxysporum* infection in the susceptible line (Figure 4G), suggesting that it may play a negative role in Fusarium wilt resistance. Additionally, Vradi01g02480 and Vradi10g07710 caught our attention for their functions related to “plant-pathogen interaction” (Supplementary Table 10).

Overall, all these candidate genes may be related to Fusarium wilt resistance, and their functions related to this resistance should be further investigated.

## Conclusion

In conclusion, 453 genes associated with Fusarium wilt resistance were identified by differential expression analysis and WGCNA. We further selected 27 candidate genes that may be involved in Fusarium wilt resistance. The present study provides insight for future research on Fusarium wilt resistance and can aid in developing strategies to combat Fusarium wilt disease in mung bean breeding.

## Data Availability Statement

The datasets presented in this study can be found in online repositories. The names of the repository/repositories and accession number(s) can be found below: NCBI BioProject repository under the accession PRJNA715596.

## Author Contributions

ZZ, JW, and YC planned and designed the experiments. ZZ, SS, and FS contributed to the lines breeding. FS performed the experiments of *F. oxysporum* resistance evaluation. YC analysed the data, validated the results by qRT-PCR, and wrote the manuscript. JW, ZZ, SS, and LW revised the manuscript. All authors contributed to the article and approved the final manuscript.

## Conflict of Interest

The authors declare that the research was conducted in the absence of any commercial or financial relationships that could be construed as a potential conflict of interest.

## Footnotes

1 http://kobascbi.pku.edu.cn
