# Supplementary material for: Transcriptome Analysis of Resistance to Fusarium Wilt in Mung Bean (Vigna radiata L.)
Source: Front Plant Sci. 2021 Jun 17;12:679629. doi: 10.3389/fpls.2021.679629 (PMC8249807; doi:10.3389/fpls.2021.679629)
Supplement: Supplementary file 1 [file Data_Sheet_1.docx]

Supplementary Figures


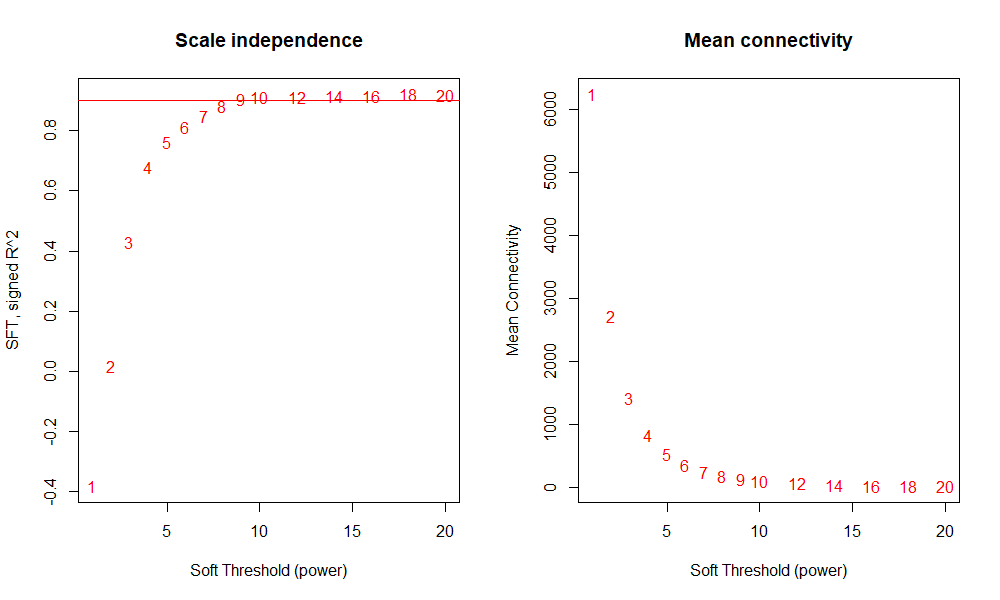


**Supplementary Figure 1** **Soft threshold determination of WGCNA.**

**
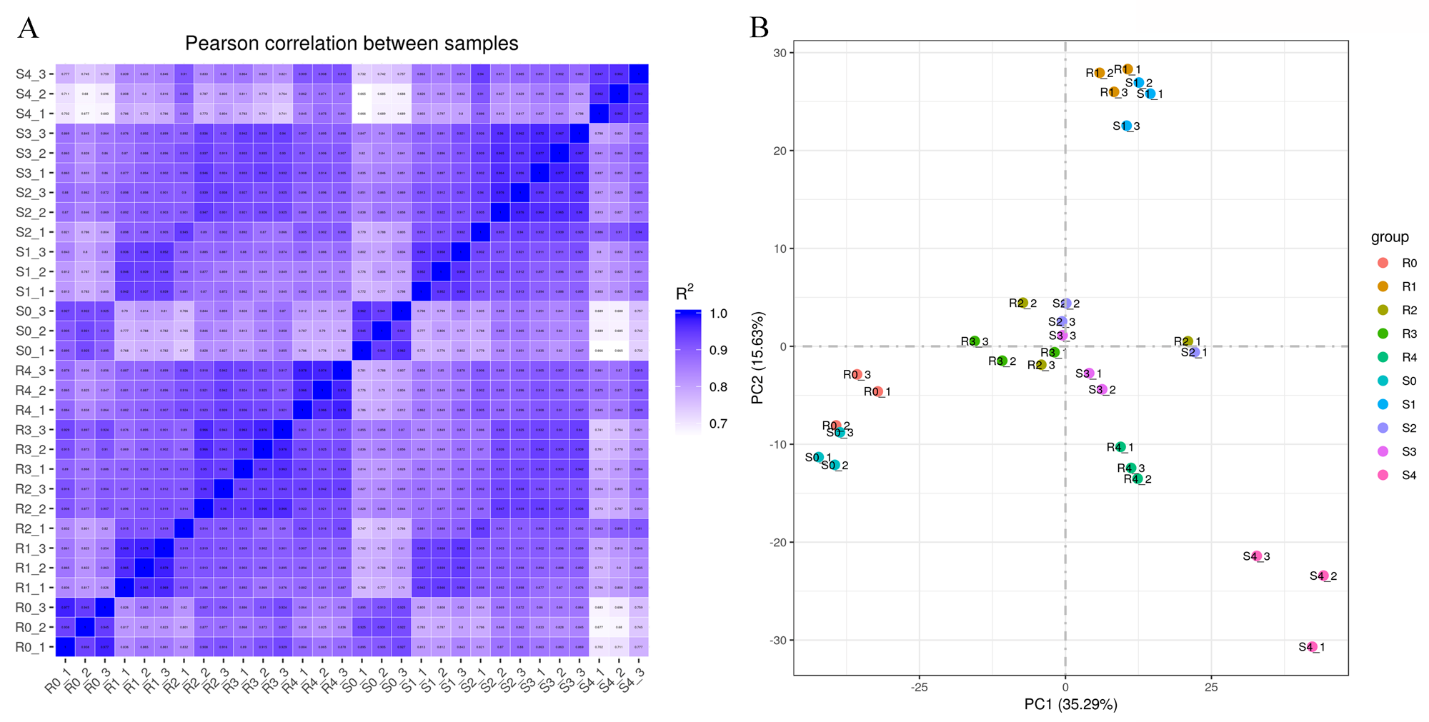
**

**Supplementary Figure 2 Correlation analysis (A) and principal component analysis (PCA, B) of samples.**
